# Case report of anosognosia for hemiplegia: A fMRI study

**DOI:** 10.1097/MD.0000000000032526

**Published:** 2022-12-30

**Authors:** Caterina Formica, Simona De Salvo, Francesco Corallo, Desiree Latella, Katia Mìcchia, Lilla Bonanno, Angelo Quartarone, Silvia Marino

**Affiliations:** a IRCCS Centro Neurolesi “Bonino Pulejo”, Messina, Italy.

**Keywords:** anosognosia for hemiplegia, motor planning, neuroimaging, stroke

## Abstract

The aim of this study is to verify the functional activation in a patient with anosognosia for hemiplegia following left brain lesions.

**Methods:**

We report a case of a 53-year-old right-handed female patient. She came to our rehabilitative unit with a diagnosis of an ischemic major stroke in the left internal carotid artery and important hemiplegia to the right side. She underwent functional magnetic resonance imaging (fMRI), during which she performed a motor imagery task.

**Results:**

The fMRI assessment showed an ischemic lesion in the frontotemporal and insular left areas. In the fMRI experiment, we revealed activation of the residual neural patterns of both hemispheres.

**Conclusion:**

We underlined an interest in the compensation mechanism that involved neural networks near brain lesions and some areas of the contro-lesional hemisphere, suggesting that the synaptic plasticity permitted an intra and inter-hemispheric reorganization of the cerebral system.

## 1. Introduction

Anosognosia (from the Greek: *a*- without, *nosos*- illness, *gnosis*-knowledge) is a neurologic phenomenon characterized by unawareness of hemiplegia.^[1]^ Stroke leads to motor deficits due to brain damage; stroke patients could believe that they can move their limbs normally.^[2]^ Studies reported the prevalence of anosognosia for motor impairment from 33% to 58% in stroke patients.^[3]^ Other studies ranged from 20% to 44% depending on the period of brain injury.^[4]^ Authors described the phenomenon as reversible within the first 3 months following the stroke.^[3,5]^ Unawareness is a common aspect of other disease patterns, such as blindness from cortical lesions, hemianopia, Korsakoff’s syndrome or basal forebrain lesions, aphasia, and other cognitive deficits. A late diagnosis often interferes with medical care and in some cases early intervention is important.^[6]^ A rehabilitative program is difficult to realize when 1 is unaware of it,^[7]^ so might not be successful.^[8]^ In a healthy person when is given a motor command, this is decoded by the language systems that activate the motor action-intentional systems, which include the premotor systems. These premotor systems not only activate the motor system but also activate a representation of how the body position will change. When the movement occurs, the afferent neurons feedback that a movement has proven. When there is a lesion in the motor systems that involve upper motor neuron or corticospinal tract or lower motor neurons or motor roots, et cetera, the feedback does not match the body representation and the patient becomes aware of disorders. According to this model, anosognosia might be associated with deficits of the action-intentional system; feedback errors due to inattention; dysfunction of body representation inducing personal neglect, but also an inability to detect a mismatch between the expected and real movement; a disconnection between the body representation and the language areas might produce verbal confabulation.

There are different hypotheses about the etiology of anosognosia for hemiplegia (AHP). According to Weinstein & Kahn the motivation to deny illness and disability exists in everyone; they postulated that unawareness of disease, including hemiplegia, was psychologically motivated denial, an unconscious defence mechanism that attenuates the potential distress of a catastrophic event such as hemiplegia.^[9]^ Weinstein & Kahn tested the denial hypothesis by ascertaining each stroke patient’s premorbid personality characteristics from relatives and close associates. They found that before their illness, patients who demonstrated anosognosia used denial as a coping strategy more frequently than did patients who were aware of their deficits. Another reason that patients with anosognosia for hemiplegia might be unaware of their hemiplegia is that they do not get the sensory feedback that the limb is weak.^[10]^ Sensory defects might have contributed to their unawareness. The phantom limb movement theory suggests that it is possible that some subjects do not perceived that their arm is paralyzed because they actually feel the limb moving. The neuropsychological model considered the anosognosia as a consequence of a cognitive impairment. In fact, there were studies that demonstrated the association between cognitive deficits and awareness of motor impairment.^[10]^ Other neuropsychological theory associated the awareness of motor impairment with a specific cognitive deficits domain such as related to a memory impairment, or the capacity to transfer new information from the working memory to the long-term memory magazine.^[5]^ Heilman developed the feedforward theory that assumes the mechanism of anosognosia as a discrepancy between the expectation of movement and the perception of the movement itself. As long as the limb movement is not requested, the effects of the injury cannot be observed. Neuroanatomical and clinical studies reported damage of fronto-temporo-parietal areas, insula and basal ganglia.^[4,11]^ Berti et al showed that in anosognosic patients the overlap of brain lesion was on the dorsal premotor cortex in 94% of patients with anosognosia and neglect, the somatosensory area, and the primary motor cortex. Both groups (patients with anosognosia and neglect/ without anosognosia and with neglect) were involved in the inferior parietal lobe, tipically associated with spatial neglect. A substantial difference between the 2 groups was that patients with pure anosognosia had largely brain damage overlaps with areas that distinguished the 2 groups.^[12]^ Karnath et al illustrated that the area specifically related to anosognosia for hemiplegia/hemiparesis is the right posterior insula. The authors showed that the 62% patients with anosognosia involved the posterior insula than in patients without anosognosia. A meta-analysis highlighted the presence of a neural network disconnection that involved the frontal, temporal, and parietal lobes, as well as insular cortex and subcortical regions in the basal ganglia and thalamus.^[13]^ Moreover, the lesions are located in the middle cerebral artery, deep and superficial showing also a disconnection of descending motor pathways.^[14]^ Two studies on voxel- based lesion analysis confirmed that anosognosia for hemiplegia reflects a multi-component disorder due to lesions affecting a distributed set of brain regions, which can lead to several co-existing deficits in sensation, attention, body representations, motor planning, memory, and even affective processing.^[15,16]^ In line, Fotopoulou and collegous, proposed a network view in which cognitive system, in addition with sensory and motor systems, contributed to the disorder.^[17]^ A functional magnetic resonance imaging (fMRI) study investigated the neural activity of brain lesions in patients with AHP during the execution of a motor task.^[18]^ The authors found that the lack of motor awareness triggered cortical regions involved in motor control in the left intact hemisphere and in the spared motor regions of the right hemisphere; patients without anosognosia did not present the same degree of activation.^[18]^ Studies showed a trend toward a higher frequency of anosognosia after right brain lesions, while the presence of this symptoms after left brain damage ranged about 49% to 86%.^[19]^ Few studies reported the unawareness of motor impairment after brain lesion on the left side.^[20]^ A possible reason is a methodological bias. In fact, the usual methods to assess anosognosia of motor impairment are structured, verbal interviews.^[21]^ Assessment that required language skills cannot be used with patients with left damage and consequent language deficits. Stone et al^[22]^ showed that 48% of aphasic patients was excluded and in Cutting’s^[3]^ study 60% of patients. Studies that discussed the relation between anosognosia for motor deficits in patients with left brain damage and functional results are few. A stroke rehabilitation study described a sample of 29 hemiplegic patients with right hemisphere damage and 17 stroke patients with left brain damage.^[23]^ Results showed that the group with a right brain injury had large lesions involving frontal, parietal and temporal cortex and evident sensory and spatial-attention impairment, while the group with left brain injury and AHP, had more detailed and small subcortical areas involved without sensory and attentional deficits.

We described an unusual case of AHP in a female patient. The unawareness of motor impairment was the results of an ischemic lesion that involved the left hemisphere. The case could contribute to the few literature evidence to verify the functional neural activation involved during a fMRI experiment of motor imagery task in a patient with AHP.

## 2. Case report

**Figure 1.**
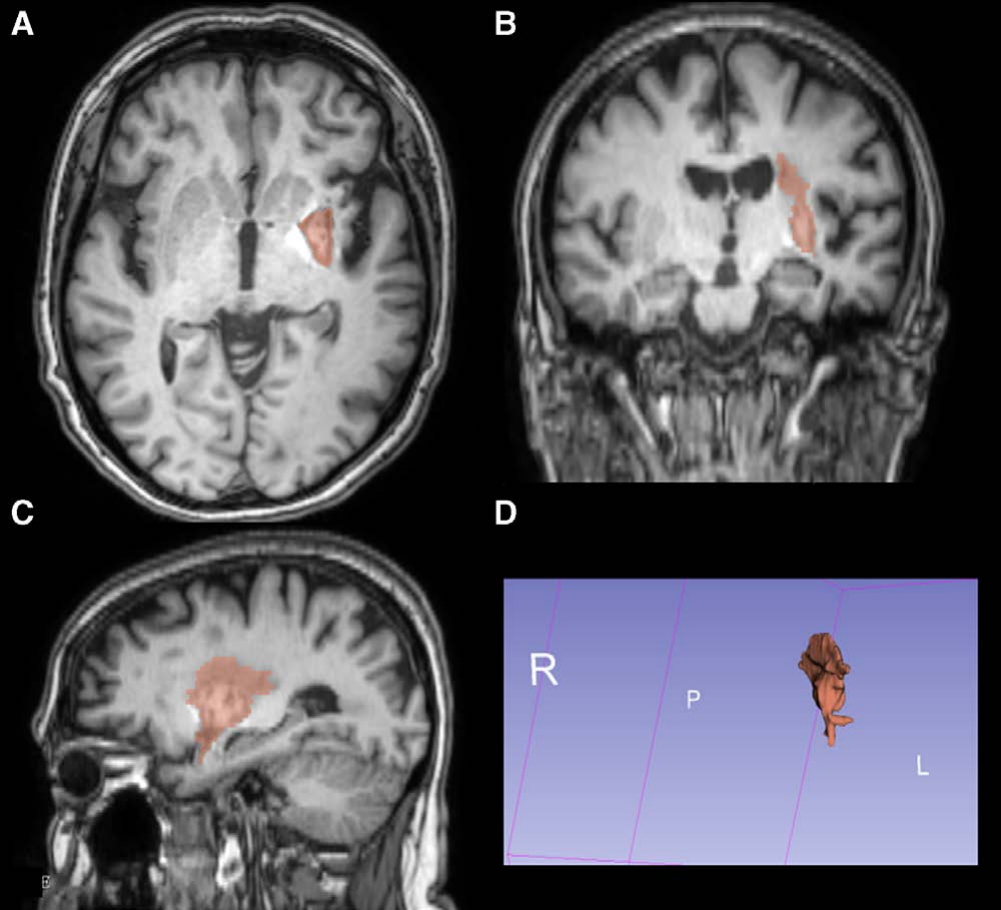
In FLAIR sequence. (A). Axial orientation; (B). Coronal orientation; (C). Sagittal orientation; (D). Segmentation of lesion.

The patient is a 53-years-old, right-handed female patient, from Sicily (southern Italy) with a graduate degree in theology and she worked as a religion teacher in a secondary school. She was married and had 2 daughters. She spoke only the Italian language. The patient suffered from arterial hypertension for about 10 years and hyperinsulinism, she also presented a patent foramen ovale of 5 millimeters and a mild mitral failure. On 2 June 2019, after getting up at 6:00 in the morning in conditions of well-being she had an onset of difficulty in speech and hypesthesia to the right upper and lower limbs. After the onset of the symptoms, the patient was admitted to the hospital, where was submitted to a brain fMRI performed with angiography showing no blood flow at the level of left internal carotid artery, and the middle cerebral artery of the left side. After this investigation a diagnosis of stroke was performed. She was transferred immediately to the Stroke Unit and was submitted to endovenous thrombolysis, and then to thromboaspiration and complete intracranial recanalization. Figure 1 showed a cerebral lesion with a volume of 7,15 cm. After 10 days, she was hospitalized to our rehabilitative unit with a diagnosis of ischemic major stroke in the left internal carotid artery and consequent hemiplegia to the right side. Neurologic examination revealed hemiplegia involving the upper and lower right limbs. Vigilant and conscious patient, collaborating to the medical examination, good understanding and execution of simple orders (close the eyes, stick out the tongue), discreetly oriented in space/time. Babinski’s sign is evocable on the right. The upright station is maintained only with support and help of an operator, gait not present, the supine postural transition from sitting to bed performed with help, transfer of bed-chair position and vice versa performed with the use of left for the person or 2 operators. Presence of bladder catheter. Patient presented a right facial hemiparesis. There was asymmetry, with the right corner of the mouth bent downward. The assessment of dysarthria revealed poor pneumo-phonatory coordination. The patient was hospitalized for 2 months in our unit to perform a rehabilitative program that included: sessions of 50 minutes of physiotherapy every day and speech therapy treatments that she performed every day to improve facial symmetry and pneumo-phonatory coordination for 2 months. The ethical permission was not applied because it was a case report, a written informed consent was obtained from the patient.

## 3. Motor and logopedic assessment

The patient’s motor functioning was evaluated using the trunk control test assesses motor impairment and predicts the recovery of walking ability. The test is done by the patient lying on the bed making four actions: roll to the weak side; roll to the strong side; balance in a sitting position on the edge of the bed with the feet off the ground for at least 30 seconds; sit up from lying down.^[24]^ Deficits of motor ability performance in activities of daily living were assessed by the Barthel Index.^[25]^ Ten variables describing activities of daily living and mobility are scored, a higher score indicates greater independence following hospital discharge (maximum 100), while a lower score indicates complete dependency (minimum of 0) (see Table 1). The motricity index evaluates the ability to perform movements with both limbs, useful in activities of daily life. It concerns grading strength based on a patient’s ability to activate muscle group, move a limb segment through a range of motion, and resist force. For the upper extremity tests, it is required to perform 3 actions: pinch grasp, elbow flexion, and shoulder abduction. While for the lower extremity test, it is required to perform hip flexion, knee extension, and dorsiflexion. The total score for each limb yes gets as a sum of the scores of the partial tests. The maximum score is assigned by default to the limb preserved.^[26]^ The berg balance scale consists of a set of 14 items that assessed static balance and falls risk in adults. The score ranges from 0 to 56. Total score can indicate a low fall risk (score 41–56), medium fall risk (score 21–40), or high fall risk (score 0–20).^[27]^ The sunnybrook facial grading system^[28]^ is a composite of 3 domains (face at rest, voluntary motion, synkinesis), 3 facial regions at rest (eye, cheek, mouth), and 5 facial regions in voluntary motion with or without synkinesis (brow lift, gentle eye closure, snarl, open mouth smile, and lip pucker). Within each domain and facial region there are 3 to 5 levels. The system generates a composite score that describes the overall static and dynamic condition of the face. For the evaluation of language skills, the patient was submitted to the aachener aphasie test ^[29]^ was administered, to evaluate if the language skills were preserved. The aachener aphasie test was divided in 6 subtests, looking at the phonology, language spontaneous, reading and writing, naming, and comprehension. (see Table 2).

**Table 1:** Scores of the motor abilities measures.

Tests	Adjusted Scores	cutoff
TCT	75	Normal
BI	15*	Deficit
BBS	31	Deficit
*MI*	*32*	Deficit

BBS = berg balance scale, BI = barthel index, MI = motricity index, TCT = trunk control test.

**Table 2:** Scores of the language abilities measures.

Tests		Adjusted scores	Level of functioning
SFRS		47*	Borderline
AAT	Token test	58	Mild
	Repetition	55	Mild
	Written language	*55*	Mild
	Naming	*50*	Mild
	Comprehension	*60*	Mild

AAT = aachener apahasia test, SFRS = sunnybrook facial rating scale.

### 3.1. Neuropsychological assessment

The patient, after 10 days post stroke, was submitted to a neuropsychological assessment (Table 3). The evaluation included tasks about executive function, short term memory and delayed recall, verbal fluency, tasks that indagated the awareness of motor impairment.In particular, we administered the montreal cognitive assessment to have a global cognitive profile in which we performed memory skills, verbal fluency, and attention.^[30]^ The executive function was assessed by the Wisconsin Card Sorting Test (WCST) related to flexible strategy and inhibition^[31]^ and the behavioral assessment of the dysexecutive syndrome related to goal-oriented planning, generation, sustaining set maintenance, self-monitoring,^[32]^ (see Table 4)The visual-analogue test assessing anosognosia for motor impairments^[33]^ was composed of 12 items, presented in a verbal and visual way, the stimuli represented daily living bimanual or bipedal actions such as “tying a knot” or “walking upstairs.” The responses were structured in a Likert scale from 0 “No Problem” to 3 “ More Problem,” in trying to perform each action. The test was completed also by the eldest daughter with the physiotherapist. The caregiver’s response is the sum of the scores obtained of the functional motor ability of the patient. The scores were compared with the patient’s responses and the discrepancy from both scores had a potential range of -36 to + 36, where zero represents total agreement (see Table 3). The patient was cooperative, partially oriented in time, well oriented in space. Spontaneous speech is slow but appropriate to the context. The neuropsychological evaluation showed a cognitive profile below normal. Executive functions are impaired, such as goal-oriented planning, flexible strategy generation, sustaining set maintenance, self-monitoring, and inhibition. From the clinical observation was emerged behavioral and emotional inadequacy, in particular, the patient showed stenosis, amimical facial expression with poor verbal initiative. All these tests were performed to assess the specific cognitive domains and to clarify the neuropsychological status of the patient.

**Table 3:** Scores of the cognitive abilities measures.

Tests		Adjusted scores	cutoff
MOCA		19*	26
WCST	Global score	68.10	90.50
	Perseverative errors	75*	42.60
	No perseverative errors	4.8	29.90
VATAm	Global score	18*	6.2
	self-evaluation VATAm (arm)	9*	3.7
	self-evaluation VATAm (leg)	6*	3.4

MOCA = montreal cognitive assessment, VATAm = visual analogue test for anosognosia for motor impairment, WCST = wisconsin card sorting test.

**Table 4:** Scores of executive functioning.

Test	Subscale	Scores	Level of functioning
BADS	Rule shift cards	1*	Deficit
	Action program	4	Normal
	Key search	2*	Borderline
	Temporal judgment	4	Normal
	Zoo map	1*	Deficit
	Modified 6 elements	1*	Deficit
	Total BADS	80*	Borderline

BADS = Behavioral Assessment of Dysexecutive Syndrome.

We reported the patient’s claims concerning her motor deficit as following:

Doctor: `Do you have any difficulty in drinking from a glass with her right hand?’

P.: `not very much.’

Doctor: `Do you have difficulty clapping your hands?’

P.: `Yes, I do.’

Doctor: `Do you have difficulty in washing your hands?’

P.: `not very much difficulty.’

## 4. Brain imaging

The patient was submitted to a 3T fMRI scanner (Achieva, Philips, Best, The Netherlands) using a 32-channel SENSE head coil. The imagines were evaluated by neuroradiologist of the hospital who described the location of the brain injury.

### 4.1. fMRI experimental procedure

MRI sequences acquired were: T1-weighted [TR = 8ms, TE = 4ms, slice thickness/gap = 1/0 mm, number of slices = 173, field of view 240 mm] used as structural reference for fMRI acquisition. fMRI-sequences were built on block paradigm of 36 volumes (number of slice = 45, slice thickness = 3 mm; TR = 4000 ms; TE = 30 ms; FOV = 224 × 240 mm; matrix = 2 × 2 mm, duration = 2.24 minutes).

The fMRI acquisition is based on a block paradigm that alternates 3 periods of activity of 16 seconds with 3 periods of rest of 8 seconds. We acquired 3 fMRI sequences. MRI experiment included a motor imagery task that involved the capacity to imagine a movement with left and right hand alternated with rest periods. Motor imagery was a technique used as a complementary method for learning motor skills, hereafter, it has been shown that training based on imagination has similar effects to real motor training.^[34]^ These results proved the mental repetition of a movement activates the same cortical areas of a real performance. Based on these studies,^[18,35]^ we used motor imagery task to observe the brain regions that were activated in a subject with anosognosia and hemiplegia after ictus. Moreover, our fMRI experiment aims to identify the systems of brain compensation and cerebral reorganization. Initially in a slide are reported the instructions.

**Figure 2.**
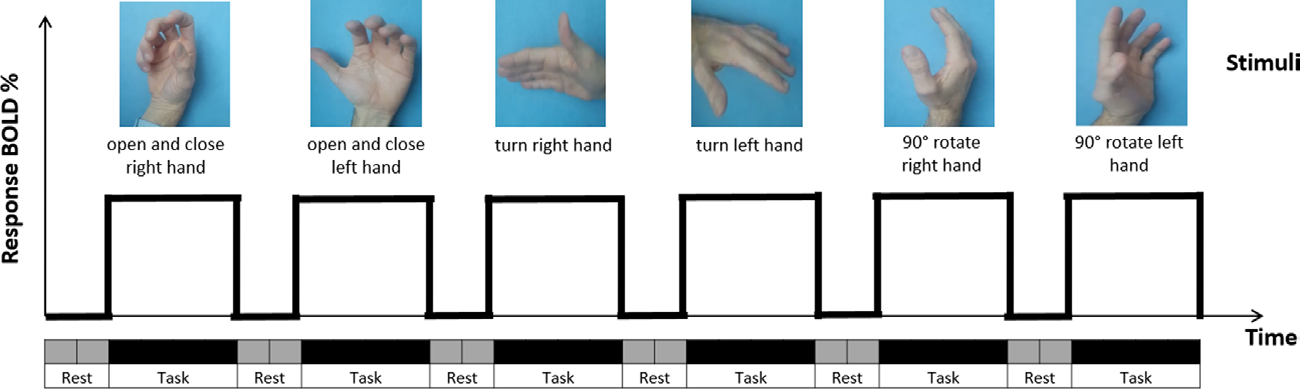
Brain mapping. Paradigm protocol of stimuli presentation.

During the task blocks, in each block a video is presented in which a hand is seen making certain movements, these movements vary from 1 task block to another (Fig. 2). The patient watching the video has to imagine doing the same movement with the corresponding hand. These task blocks alternate with rest blocks in which the patient is shown a blank screen and does not have to think or do anything.

### 4.2. fMRI analysis

FSL (FMRIB’s Software Library, www.fmrib.ox.ac.uk/fsl) was used for fMRI analysis.

Motion correction using MCFLIRT,^[36]^ scalp removal using BET,^[37]^ spatial smoothing using a Gaussian kernel of FWHM = 6 mm, mean-based intensity normalization of all volumes by the same factor and highpass temporal filtering (sigma = 30 s) were the pre-processing procedures. Registration of functional images to high resolution structural images was performed with FLIRT.^[36,38]^ For the analysis, we used the time course of motor task as the main explanatory variable (EV) convolved with a Double-Gamma hemodynamic response function. It is a mixture of 2 Gamma functions - a standard positive function and a small delayed, inverted Gamma to model the late undershoot. he resulting activation maps were normalized via non-linear registration of the MPRAGE to the MNI (Montreal Neurological Institute) 2-mm brain template and applying a cluster significance threshold of Z > 2.3 and a (corrected) cluster significance threshold of *P* < .05.

## 5. Results

The psychological assessment showed symptoms typical of a right brain lesion. We observed behavioral changes about lack of verbal initiative, apathy, lack of emotional expression and AHP on the right hand. Patient’s visual-analogue test assessing anosognosia for motor impairments scores reported an overestimation of her movement abilities. The same questions were proposed to the caregiver about the ability of the patient and she highlighted that the patient was unable to perform many of the activities that the patient claimed to be able to do. From these test scores emerged the real and objective motor skills of the patient, indicating a greater dependence in daily living activities, these results correlated with Barthel Index score (Table 1). The fMRI showed an ischemic lesion in the frontotemporal and insular left areas.

**Figure 3.**
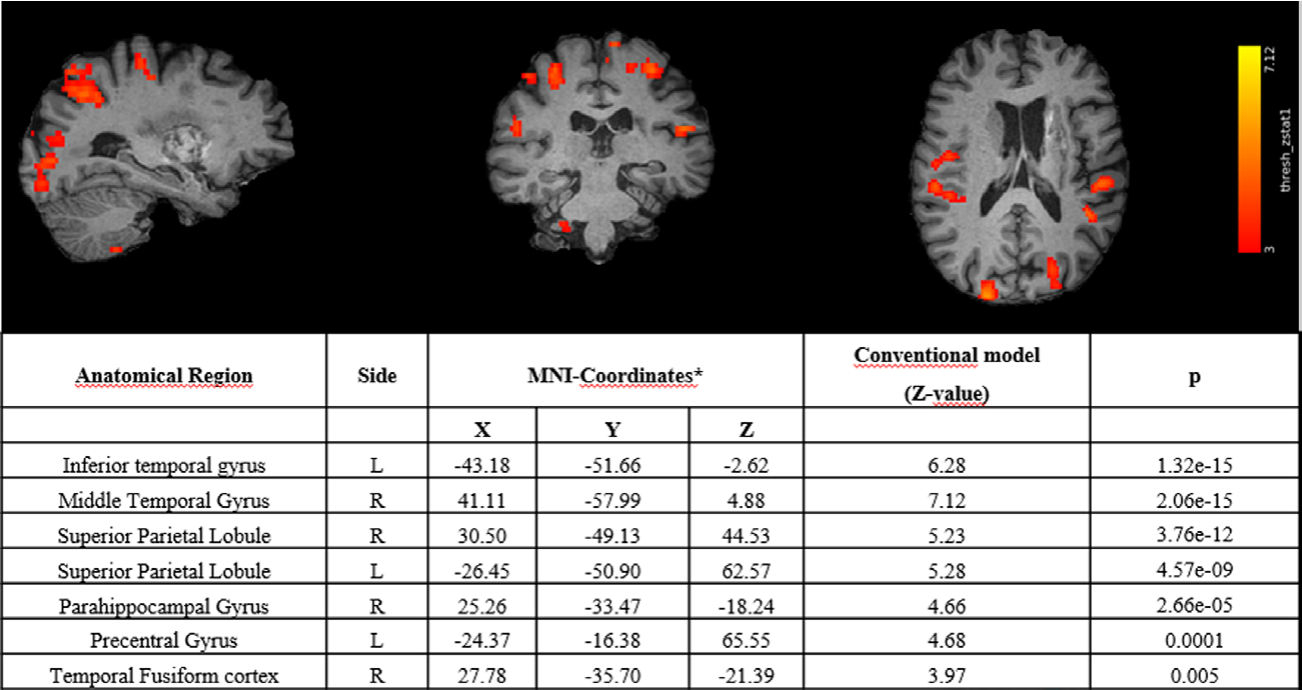
Cerebral activation fMRI analysis during a video sequence thinking about moving the corresponding hand. fMRI = functional magnetic resonance imaging.

During the task, we revealed a more functional activation in bilateral side with statistical threshold (*P* < .001) (Fig. 3). In particular, we highlighted 3 Region of Interest (ROI) in left side (Fig. 3): inferior temporal gyrus (Z = 6.28; *P* < .001), in superior parietal lobule (Z = 5.28, *P* < .001) and in precentral gyrus (Z = 4.68;*P* = .0001). In the right side we showed 4 ROI (Fig. 3): middle temporal gyrus (Z = 7.12;*P* < .001), superior parietal lobule (Z = 5.23;*P* < .001), para-hippocampal gyrus (Z = 4.66;*P* < .001) and temporal fusiform cortex (Z = 3.97;*P* = .005).

## 6. Discussion

The most important observation in this case was the presentation of a patient with anosognosia for hemiplegia with left brain lesions without aphasia. We investigated the neuronal pattern activations during a motor imagery task and the strategic reorganization of the residual neural activity in a patient with AHP of the right upper limb following a major stroke that involved the fronto-temporal and insular left areas. During the fMRI experiment, we revealed activation of the right and left hemisphere. In particular, we highlighted activation of pre-motor area, parietal and temporal brain region of the left side, while, in the right side we showed activation of the middle temporal gyrus, the superior parietal lobe and cerebellum (Fig. 1). fMRI studies demonstrated the role of the left brain in motor planning, motor imagery and motor representation tasks.^[39,40]^ Gandola et al, showed that the illusion of moving the plegic hands is characterized by an activation of the cortical regions that were implicated in motor control in the left hemisphere, and in the motor regions of the right hemisphere, these activations were not the same in healthy patients. As mentioned above, few literature studies reported functional results.^[18]^ In particular, Hartman-Maeir et al, showed that right brain injury involved large areas of frontal, parietal and temporal cortex while, left brain injury and AHP were correlated with more detailed and small subcortical areas.^[23]^ Instead, our fMRI findings demonstrated that also extended left cerebral lesions may lead to the same outcome. Our results, in accordance with another fMRI study,^[18]^ showed the activations of the spared motor, premotor cortex, and cerebellum. In our case, we observed the same neural activation following the instruction to imagine a movement of the paralyzed hand. Therefore, we confirmed that these tasks required the activation of the same neural patterns, in both cases: when the request is the execution of a movement or simply imaging a movement. A limit of our case is the number of the sample, the absence of a control group and the lack of standardized assessment. However, a fMRI study demonstrated that the neural patterns activated, for the movement of both hands, were overlapping with the healthy subjects.^[18]^ In conclusion, our results provided evidence of the activation of motor areas in a condition of unawareness of motor impairment,^[41]^ in a patient with a left-brain lesion. This case highlighted an interest of the compensation mechanism that involved neural networks near brain lesions and some areas of the contro-lesional hemisphere suggesting that the synaptic plasticity permitted an intra and inter-hemispheric reorganization of the cerebral system^[42]^ to modulate the residual ability to generate motor planning. Our findings are consistent with Heilman’s feedforward theory, according to which anosognosia is the result of a discrepancy between the expectation of movement and the perception of the movement. In according to the neuroanatomical findings our patient showed that the area specifically related to anosognosia for hemiplegia/hemiparesis are the fronto-temporal and parietal lobes, insular region and subcortical areas.^[13,14]^

## Author contributions

**Conceptualization:** Caterina Formica.

**Data curation:** Simona De Salvo.

**Formal analysis:** Lilla Bonanno.

**Investigation:** Desiree Latella, Katia Mìcchia.

**Methodology:** Francesco Corallo.

**Visualization:** Angelo Quartarone.

**Writing – review & editing:** Silvia Marino.
